# Functional Carbon Capsules Supporting Ruthenium Nanoclusters for Efficient Electrocatalytic ^99^TcO_4_^−^/ReO_4_^−^ Removal from Acidic and Alkaline Nuclear Wastes

**DOI:** 10.1002/advs.202303536

**Published:** 2023-09-10

**Authors:** Xiaolu Liu, Yinghui Xie, Yang Li, Mengjie Hao, Zhongshan Chen, Hui Yang, Geoffrey I. N. Waterhouse, Shengqian Ma, Xiangke Wang

**Affiliations:** ^1^ College of Environmental Science and Engineering North China Electric Power University Beijing 102206 P. R. China; ^2^ MacDiarmid Institute for Advanced Materials and Nanotechnology School of Chemical Sciences The University of Auckland Auckland 1142 New Zealand; ^3^ Department of Chemistry University of North Texas Denton TX 76201 USA

**Keywords:** adsorbent‐electrocatalyst, environmental remediation, perrhenate, pertechnetate, ruthenium clusters

## Abstract

The selective removal of the β‐emitting pertechnetate ion (^99^TcO_4_^−^) from nuclear waste streams is technically challenging. Herein, a practical approach is proposed for the selective removal of ^99^TcO_4_^−^ (or its surrogate ReO_4_^−^) under extreme conditions of high acidity, alkalinity, ionic strength, and radiation field. Hollow porous N‐doped carbon capsules loaded with ruthenium clusters (Ru@HNCC) are first prepared, then modified with a cationic polymeric network (R) containing imidazolium‐N^+^ units (Ru@HNCC‐R) for selective ^99^TcO_4_^−^ and ReO_4_^−^ binding. The Ru@HNCC‐R capsules offer high binding affinities for ^99^TcO_4_^−^/ReO_4_^−^ under wide‐ranging conditions. An electrochemical redox process then transforms adsorbed ReO_4_^−^ to bulk ReO_3_, delivering record‐high removal capacities, fast kinetics, and excellent long‐term durability for removing ReO_4_^−^ (as a proxy for ^99^TcO_4_^−^) in a 3 m HNO_3_, simulated nuclear waste‐Hanford melter recycle stream and an alkaline high‐level waste stream (HLW) at the U.S. Savannah River Site (SRS). In situ Raman and X‐ray absorption spectroscopy (XAS) analyses showed that adsorbed Re(VII) is electrocatalytically reduced on Ru sites to a Re(IV)O_2_ intermediate, which can then be re‐oxidized to insoluble Re(VI)O_3_ for facile collection. This approach overcomes many of the challenges associated with the selective separation and removal of ^99^TcO_4_^−^/ReO_4_^−^ under extreme conditions, offering new vistas for nuclear waste management and environmental remediation.

## Graphical abstract

Hollow carbon capsules supporting ruthenium metal clusters and a thin polymer layer rich in imidazolium‐N^+^ units enable highly efficient ^99^TcO_4_^−^/ReO_4_^−^ removal under harsh conditions relevant to nuclear fuel waste processing and storage. The imidazolium‐N^+^ promotes selective ^99^TcO_4_^−^/ReO_4_^−^ binding, while the Ru clusters provide a reversible electron transfer platform for electrocatalytic conversion of ^99^TcO_4_^−^/ReO_4_^−^ to TcO_3_/ReO_3_, resulting in record‐high extraction efficiencies.

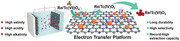

## 1 Introduction

Reducing anthropogenic CO_2_ emissions by investment in non‐fossil fuel‐based energy technologies and infrastructure is vital to mitigating the worst effects of global warming.^[^ ^1^ ^]^ For many decades, nuclear fission reactors have offered a low carbon source of electricity.^[^ ^2^ ^]^ However, to ensure the sustainability and safety of the nuclear energy sector, simple and economically viable technologies must be developed for the recovery and separation of fissionable nuclides and long‐lived toxic fission products from nuclear wastes.^[^ ^3^ ^]^ Nuclear waste contains unspent uranium‐235 (^235^U), large amounts of non‐fissile uranium‐238 (^238^U), plutonium‐239 (^239^Pu), along with technetium‐99 (^99^Tc), and other radionuclides produced through nuclear reactions, all of which are typically dissolved in aqueous 3 m HNO_3_ during the first stage of nuclear waste management.^[^ ^4^ ^]^ The recovery of uranium to be fed back into fuel manufacture is an important step in the nuclear fuel cycle. However, technetium‐99 (^99^Tc, a β‐emitting isotope generated from the fission of ^235^U), present in the form of ^99^TcO_4_^−^, interferes with valence state control during the uranium extraction and recovery process, creating a technical bottleneck.^[^ ^5^ ^]^ This motivates the development of adsorbent technologies for the selective removal of ^99^TcO_4_^−^ from uranium resources during the concentrated nitric acid solution processing stage and before the plutonium uranium redox extraction (PUREX) process. However, selective capture of ^99^TcO_4_^−^ is hampered by the extreme nature of nuclear waste streams, which are typically strong acidic, possess high ionic strength, and have a highly ionizing radiation field.^[^ ^4a,4b^ ^]^ In addition to the acidic streams in the PUREX process, there are also millions of gallons of low‐level radioactive waste (LAW) stored at the Hanford site in Washington State and alkaline high‐level radioactive waste (HLW) at the U.S. Savannah River Site (SRS) in South Carolina.^[^ ^6^ ^]^ Due to its toxicity, high solubility, and environmental mobility, ^99^TcO_4_^−^ leaks from nuclear waste processing or storage sites into groundwater would cause serious environmental contamination, necessitating techniques that could efficiently capture ^99^TcO_4_^−^ in the event of a leak.^[^ ^6^, ^7^ ^]^

To address the above issues, various techniques including solvent extraction,^[^ ^8^ ^]^ ion exchange,^[^ ^9^ ^]^ photocatalysis,^[^ ^10^ ^]^ electrocatalysis,^[^ ^11^ ^]^ and chemical catalysis^[^ ^12^ ^]^ have been developed for the extraction of ^99^TcO_4_^−^/ReO_4_^−^ under neutral, mildly acidic or alkaline conditions. However, few of these methods can efficiently remove ^99^TcO_4_^−^ under conditions relevant to real radioactive waste, such as in 3 m HNO_3_ or super alkaline conditions, at high ionic strengths or following large doses of radiation, motivating the search for more practical ^99^TcO_4_^−^ capture technologies.

Porous carbon‐based materials find widespread application in chemical catalysis,^[^ ^13^ ^]^ energy storage,^[^ ^14^ ^]^ oxygen reduction reaction,^[^ ^15^ ^]^ electro‐Fenton reactions,^[^ ^16^ ^]^ and many other processes.^[^ ^17^ ^]^ Recently, we pioneered an adsorption‐electrocatalytic method for the extraction of uranium from seawater using amidoxime‐functionalized metal‐nitrogen‐carbon materials.^[^ ^18^ ^]^ The surface amidoxime groups exhibited strong binding affinity toward UO_2_^2+^ over other metal cations, while the metal single atoms anchored on the hierarchically porous N‐doped carbon support enabled fast electrocatalytic conversion of adsorbed UO_2_^2+^ to solid Na_2_O(UO_3_·H_2_O)_x_ through a reversible single electron transfer process. We hypothesized that a similar general approach could be used for the selective capture of ^99^TcO_4_^−^ (and its surrogate ReO_4_^−^), motivating a detailed investigation.

**Figure 1:**
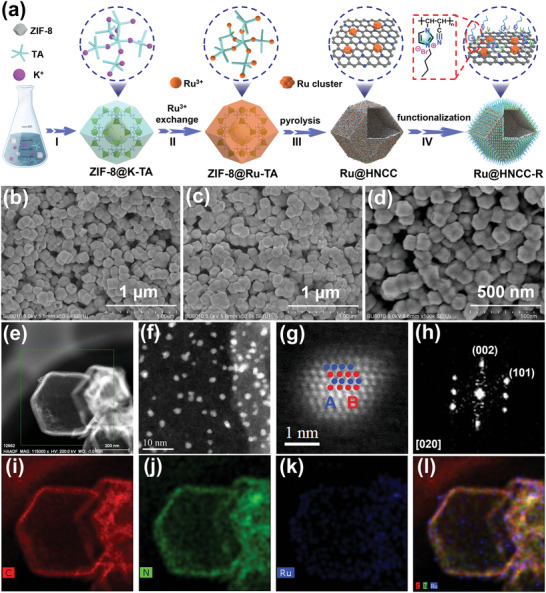
a) Schematic illustration of the synthesis of Ru@HNCC‐R. b–d) SEM images of ZIF‐8@K‐TA, ZIF‐8@Ru‐TA, and Ru@HNCC, respectively. e,f) HAADF‐STEM images of Ru@HNCC. g,h) Aberration‐corrected HAADF‐STEM images of an individual Ru cluster and its corresponding FFT pattern. i–l) Corresponding EDS maps reveal a homogeneous distribution of C (red), N (green), and Ru (blue) over the carbon capsules.

Herein, we describe a novel adsorption‐electrocatalytic system for the selective separation and recovery of ^99^TcO_4_^−^ from 3 m HNO_3_, Hanford LAW and SRS HLW radioactive wastes. We aimed to prepare an adsorbent‐electrocatalyst rich in cationic sites with a high chelating affinity for ^99^TcO_4_^−^, thus realizing selective and fast adsorption of ^99^TcO_4_^−^ with the assistance of an applied electric field. Subsequently, the adsorbed ^99^TcO_4_^−^ was then to be transformed into a solid product for collection. Our strategy involved the synthesis of ruthenium clusters supported on hollow nitrogen‐doped carbon capsules (denoted as Ru@HNCC), which were then functionalized with a cationic polymeric network (R) containing imidazolium‐N^+^ units, yielding Ru@HNCC‐R (**Figure** **1**a). The imidazole cation sites imparted the capsules with relatively high hydrophilicity and a strong binding affinity to selective adsorb ^99^TcO_4_^−^ (or its surrogate ReO_4_^−^) under various conditions, which was then electrocatalytically reduced to ^99^TcO_3_ (or ReO_3_) on the surface of the capsules. In situ Raman spectroscopy and X‐ray absorption spectroscopy established that adsorbed Re(VII) was first reduced to a Re(IV) intermediate (i.e., ReO_2_), then re‐oxidized to Re(VI) in the form of ReO_3_, thereby allowing the electrochemical ReO_4_^−^/^99^TcO_4_^−^ removal process on the Ru@HNCC‐R surface to be fully understood at a molecular level. Ru@HNCC‐R offered outstanding extraction performance when tested in 3 m HNO_3_, simulated Hanford LAW and SRS HLW solutions, delivering record‐high removal capacities of 449 mg g^−1^, 403 mg g^−1^, and 219 mg g^−1^, respectively, for ReO_4_^−^ (surrogate of ^99^TcO_4_^−^) and excellent durability. To the best of our knowledge, this is the first time that an adsorption‐electrocatalysis approach has been adopted for the selective adsorption of ^99^TcO_4_^−^/ReO_4_^−^ under such extreme conditions, guiding the improved processing of nuclear waste.

## 2 Results and Discussion

### 2.1 Synthesis and Characterization of Materials

**Figure 2:**
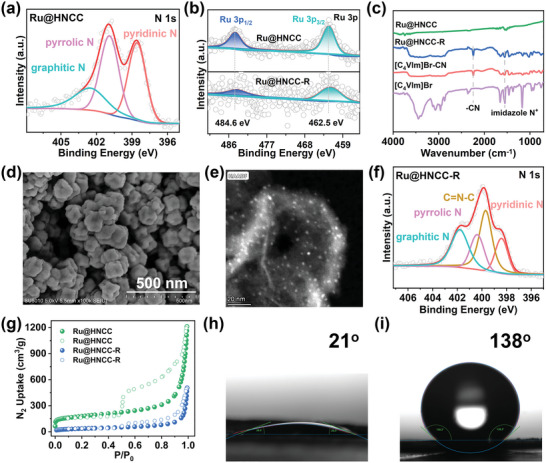
a) N 1s XPS spectrum for Ru@HNCC. b) Ru 3p XPS spectra for Ru@HNCC and Ru@HNCC‐R. c) FT‐IR spectra of different materials. d) SEM image of Ru@HNCC‐R. e) HAADF‐STEM image of Ru@HNCC‐R. f) N 1s XPS spectrum for Ru@HNCC‐R. g) N_2_ sorption isotherms for Ru@HNCC and Ru@HNCC‐R. h) Contact angle for a water droplet on a pressed pellet of Ru@HNCC‐R. i) Contact angle for a water droplet on a pressed pellet of Ru@HNCC.

As illustrated in Figure 1a, the first step (step I) in the synthesis of the Ru@HNCC‐R adsorbent‐electrocatalyst was the preparation of metal‐organic framework coated potassium‐tannic acid (ZIF‐8@K‐TA) nanocrystals.^[^ ^19^ ^]^ Exchanging the potassium cations with ruthenium cations yielded ZIF‐8@Ru‐TA (step II). Powder X‐ray diffraction (PXRD) and Fourier transform infrared spectroscopy (FT‐IR) data revealed that the ZIF‐8 core retained its crystalline structure after coating with the tannic polymer and the subsequent cation exchange step (Figures S1 and S2, Supporting Information). Scanning electron microscopy (SEM) images showed that ZIF‐8, ZIF‐8@K‐TA, and ZIF‐8@Ru‐TA all possessed the same polyhedral morphology (Figure 1b,c; Figure S3, Supporting Information). A subsequent pyrolysis treatment under an argon atmosphere converted the organic components of ZIF‐8@Ru‐TA into hollow nitrogen‐doped carbon capsules, with the adsorbed Ru(III) cations being concomitantly reduced to zero‐valent clusters, thus yielding Ru@HNCC (step III). SEM revealed that polyhedral morphology of ZIF‐8@Ru‐TA was retained on forming Ru@HNCC (Figure 1d). High‐angle annular dark‐field scanning transmission electron microscopy (HAADF‐STEM) images (Figure 1e,f) showed the presence of small metallic Ru clusters uniformly dispersed over the hollow nitrogen‐doped carbon capsules. The average diameter of the Ru clusters was determined to be ≈1.5 nm (Figure S4, Supporting Information). The atomic‐resolution HAADF‐STEM image and corresponding fast Fourier transform (FTT) pattern of an individual Ru nanoparticle revealed a hexagonal close‐packed (hcp) crystal phase:, i.e., “ABABAB…” stacking along the [020] direction (Figure 1g,h). Previous studies have shown that the hcp Ru structures demonstrate good stability and high catalytic activity in electrocatalytic redox reactions.^[^ ^20^ ^]^ The corresponding energy dispersive X‐ray spectroscopy (EDS) mapping images confirmed a uniform distribution of C, N, and Ru throughout the capsules (Figure 1i–l). No metallic Ru signals were detected by PXRD due to the small size of the clusters (Figure S5, Supporting Information). X‐ray photoelectron spectroscopy (XPS) was used to determining the surface electronic structure of the Ru@HNCC. The survey spectrum confirmed the presence of C, N, and Ru elements (Figure S6, Supporting Information), along with a small amount of O (likely from surface C─O/C═O species). The N 1s XPS spectrum revealed peaks at 398.6, 400.9, and 402.6 eV, corresponding to pyridinic N, pyrrolic N, and graphitic N, respectively (**Figure** **2**a). The data indicates that the nitrogen atoms were doped into the carbon support. The Ru 3p spectrum of Ru@HNCC was dominated by peaks at 462.5 and 484.6 eV (Figure 2b), which could readily be assigned to the Ru 3p_3/2_ and Ru 3p_1/2_ signals, respectively, of a metallic Ru species.

Subsequently, a polymeric network containing imidazole‐N^+^ cation sites was attached to the surface of Ru@HNCC (Figure 1a, step IV). This involved two steps. First, a 1‐butyl‐3‐vinylimidazolium bromide‐co‐acrylonitrile copolymer (PBA) was prepared via the copolymerization of 1‐butyl‐3‐vinylimidazolium bromide [C_4_VIm]Br and acrylonitrile in dimethyl sulfoxide (DMSO) under a nitrogen atmosphere (Scheme S1, Supporting Information).^[^ ^21^ ^]^ Immersion of Ru@HNCC in a freshly prepared PBA/DMSO solution resulted in PBA‐coated Ru@HNCC (denoted as Ru@HNCC‐R), with abundant imidazolium‐N^+^ units now on the surface of the hollow capsules. The FT‐IR spectrum of Ru@HNCC‐R showed vibrations at 2246 and 1549 cm^−1^, respectively, which could readily be assigned to C≡N and imidazolium‐N^+^ groups of the PBA polymer, verifying the successful modification of Ru@HNCC with PBA (Figure 2c).^[^ ^22^ ^]^ SEM and HAADF‐STEM images revealed that the Ru clusters remained uniformly dispersed throughout the capsules after the PBA functionalization step (Figure 2d,e). XPS confirmed that Ru@HNCC‐R contained Ru^0^ (Figure 2b), demonstrating that the PBA functionalization process did not affect the Ru valence state.^[^ ^23^ ^]^ The appearance of a new C═N─C peak at 399.7 eV, together with a decrease in the intensities of the pyridinic N and graphitic N signals, offered further evidence that Ru@HNCC‐R was an inorganic‐organic composite (Figure 2f). The Br 3d XPS spectrum showed clear signals attributed to the bromide ion in PBA (Figure S7, Supporting Information). The Raman spectra for Ru@HNCC and Ru@HNCC‐R showed signals at 1586 and 1355 cm^−1^, corresponding to graphitic and disordered carbon domains, respectively (Figure S8, Supporting Information). The I_D_/I_G_ ratio was similar for Ru@HNCC and Ru@HNCC‐R. PXRD showed the carbon support in Ru@HNCC‐R (Figure S9, Supporting Information) was amorphous and similar to that in Ru@HNCC (Figure S5, Supporting Information), showing only weak C(002) and C(100) reflections. These data confirmed that Ru@HNCC‐R consisted of N‐doped carbon capsules supporting metallic Ru clusters, coated by a thin layer of PBA rich in cyano and imidazolium‐N^+^ sites groups.

Ru@HNCC contained 1.72 wt.% Ru and 5.06 wt.% N, as determined by inductively coupled plasma optical emission spectrometry (ICP‐OES) and elemental analysis (Table S1, Supporting Information). Ru@HNCC‐R contained less ruthenium (0.79 wt.%) and more nitrogen (7.06 wt.%), consistent with the introduction of a thin PBA rich in cyano groups and imidazole‐N^+^ groups. The specific surface area and porosity of Ru@HNCC and Ru@HNCC‐R were examined by collecting N_2_ adsorption‐desorption isotherms at 77 K on degassed samples. Both samples showed typical type IV isotherms, with the Brunauer‐Emmett‐Teller (BET) surface areas of Ru@HNCC and Ru@HNCC‐R calculated to be 686 and 165 m^2^ g^−1^, respectively (Figure 2g). The lower BET surface area for Ru@HNCC‐R is attributed to the surface PBA layer partially occupying the pores in the underlying Ru@HNCC core. Next, we examined the surface wettability properties of Ru@HNCC and Ru@HNCC‐R by collecting water contact angle data. The water contact angle was ≈21° for Ru@HNCC‐R, much lower than for Ru@HNCC (≈138°), implying the PBA coating significantly improved the hydrophilicity of the capsules (Figure 2h,i). The electrical resistance of Ru@HNCC‐R was slightly higher than that of Ru@HNCC, as expected with the introduction of the organic PBA coating. Nevertheless, Ru@HNCC‐R retained excellent conductivity after functionalization with PBA (Figure S10, Supporting Information). Based on the aforementioned structural characteristics, Ru@HNCC‐R possessed all the essential components to be an effective adsorbent‐electrocatalyst for the separation and recovery of ^99^TcO_4_^−^ (and ReO_4_^−^) from nuclear wastes, as confirmed by the adsorption and adsorption‐electrocatalysis experiments below.

### 2.2 Pertechnetate and Perrhenate Physicochemical Adsorption Studies

**Figure 3:**
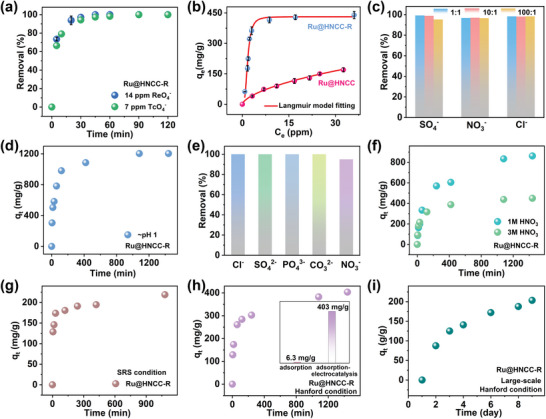
a) ^99^TcO_4_^−^ and ReO_4_^−^ adsorption kinetics on Ru@HNCC‐R at an initial ^99^TcO_4_^−^ and ReO_4_^−^ concentration of ≈7 and ≈14 ppm, respectively. b) Equilibrium adsorption isotherms for ReO_4_^−^ on Ru@HNCC‐R and Ru@HNCC, respectively. c) Effect of possible competing anions on ReO_4_^−^ uptake by Ru@HNCC‐R. d) ReO_4_^−^ extraction from aqueous solution with an initial ReO_4_^−^ concentration of ≈100 ppm, using Ru@HNCC‐R and Ru@HNCC as adsorbent‐electrocatalysts. e) Effect of possible competing anions on ReO_4_^−^ uptake by Ru@HNCC‐R via adsorption‐electrocatalysis. f) ReO_4_^−^ extraction from 1 m and 3 m HNO_3_ with an initial ReO_4_^−^ concentration of ≈100 ppm, using Ru@HNCC‐R as an adsorbent‐electrocatalyst. g,h) ReO_4_^−^ extraction under SRS and Hanford conditions, using Ru@HNCC‐R as an adsorbent‐electrocatalyst (inset shows a comparison of physiochemical adsorption performance with the adsorption‐electrocatalysis method). i) Large‐scale extraction of ReO_4_^−^ from a simulated Hanford solution by the Ru@HNCC‐R adsorbent‐electrocatalyst.

To evaluate the ^99^TcO_4_^−^ adsorption performance of Ru@HNCC‐R, we conducted preliminary physiochemical adsorption studies in ^99^TcO_4_^−^ and ReO_4_^−^ aqueous solutions. In a ≈7 ppm ^99^TcO_4_^−^ aqueous solution, Ru@HNCC‐R showed fast adsorption kinetics with a removal efficiency of 80% after 10 min and 99% after 60 min (**Figure** **3**a). As expected, the adsorption isotherm for ReO_4_^−^ (≈14 ppm) was remarkably similar to that of ^99^TcO_4_^−^, encouraging the use of non‐radioactive ReO_4_^−^ for subsequent experiments. Equilibrium adsorption experiments were next conducted by varying the initial ReO_4_^−^ concentration from 0 to 80 ppm, while maintaining an adsorbent‐to‐liquid ratio of 0.1 g L^−1^ (Figure 3b; Figure S11, Supporting Information). The adsorption capacity of Ru@HNCC‐R was determined to be 439.65 ± 17.34 mg g^−1^ for ReO_4_^−^ using a Langmuir model (Figure 3b; and Table S2, Supporting Information). In comparison, Ru@HNCC showed relatively slow adsorption kinetics and a lower adsorption capacitiy under similar conditions, demonstrating that PBA played an important role in the binding of ^99^TcO_4_^−^ and ReO_4_^−^. Considering the coexistence of huge excesses of competing anions (such as SO_4_^2−^, NO_3_^−^, Cl^−^, etc.) in nuclear wastewater, we then evaluated the adsorption selectivity of Ru@HNCC‐R toward ReO_4_^−^ in the presence of various anions. Figure 3c showed that the ReO_4_^−^ removal efficiency of Ru@HNCC‐R was not influenced by the presence of SO_4_^2−^, NO_3_^−^, Cl^−^ at the molar ratios of 1:1, 1:10, or even 1:100. Given that practical nuclear waste is either highly acidic or highly alkaline, further adsorption experiments using Ru@HNCC‐R were carried out in 3 m HNO_3_ and simulated Hanford solutions. Even under such strongly acidic conditions, removal ratios of 7.5% and 6.9% were obtained for ReO_4_^−^ and ^99^TcO_4_^−^, respectively (Figure S12, Supporting Information). Moreover, Ru@HNCC‐R is able to remove ^99^TcO_4_^−^/ReO_4_^−^ efficiently (removal ratio > 64%) from solutions relevant to LAW streams at US legacy nuclear sites (Figure S13, Supporting Information). These adsorption results confirmed that Ru@HNCC‐R offered a strong affinity for ^99^TcO_4_^−^ and ReO_4_^−^ at high ionic strengths and under extreme pH conditions.

### 2.3 Adsorption‐Electrocatalysis Studies

Encouraged by the above adsorption results, we next performed electrocatalytic ReO_4_^−^ extraction studies using Ru@HNCC‐R in simulated acidic and alkaline nuclear waste solutions. We first collected cyclic voltammetry (CV) data in 0.1 m HNO_3_ (≈pH 1) and 1 m HNO_3_ (≈pH 0) solutions containing ReO_4_^−^ ions using a traditional three‐electrode electrochemical cell. Several redox peaks were observed ≈−0.3 and 0.7 V, attributed to the reduction of Re(VII) to Re(IV) and oxidation of Re(IV) to Re(VI), respectively (Figure S14, Supporting Information).^[^ ^24^ ^]^ Similar results were obtained for ReO_4_^−^ dissolved in simulated SRS and Hanford solutions (Figure S14, Tables S3 and S4, Supporting Information). These results established that Re(VII) could be reduced to Re(IV) at negative voltages below 0 V (vs RHE), with the Re(IV) able to be reoxidized to Re(VI) at voltages above 0 V (vs RHE). On the basis of these findings, we then studied ReO_4_^−^ extraction on Ru@HNCC‐R using a square wave conversion method by alternating the voltage between −5 and 0 V at a frequency of 400 Hz.^[^ ^25^ ^]^ Carbon felt loaded Ru@HNCC‐R was employed as both the positive and negative electrodes. Ru@HNCC‐R could rapidly remove ReO_4_^−^ at ≈pH 1, resulting in a removal capacity of 1204 mg g^−1^ over 18 h (Figure 3d). We observed that the pH of the solution increased from 1 to 1.3 after the electrocatalysis, suggesting that H^+^ was consumed during the adsorption‐electrocatalysis process. Ru@HNCC showed much lower removal capacity under similar conditions. We then investigated the electrocatalytic extraction selectivity of Ru@HNCC‐R in the presence of one equivalent of potentially competing anions. The removal efficiency for ReO_4_^−^ was > 95% in the presence of the different anions (Figure 3e). Moreover, Ru@HNCC‐R could rapidly remove ReO_4_^−^ in 1 m HNO_3_ solution (containing ≈100 ppm of ReO_4_^−^), with a capacity reaching 862 mg g^−1^ for ReO_4_^−^ (Figure 3f). The electrocatalytic activity of Ru@HNCC‐R was further investigated by conducting ReO_4_^−^ extraction experiments in 3 m HNO_3_ solution, with the capacities up to 449 mg g^−1^ realized for ReO_4_^−^ (Figure 3f). The disappearance of Br signals after the extraction experiment demonstrated that Br^−^ can be fully exchanged with ReO_4_^−^ (Figure S15, Supporting Information). We next assessed the durability of Ru@HNCC‐R as an adsorbent‐electrocatalyst in 3 m HNO_3_ solutions containing ReO_4_^−^. Ru@HNCC‐R showed outstanding electrochemical activity and durability during cycling in 3 m HNO_3_, with an uptake capacity of 303 mg g^−1^ achieved after eight cycles (Figure S16, Supporting Information). SEM and TEM images showed that Ru@HNCC‐R retained its initial hollow dodecahedral capsule morphology after cycling tests in 3 m HNO_3_, implying excellent structural stability (Figure S17, Supporting Information).

**Figure 4:**
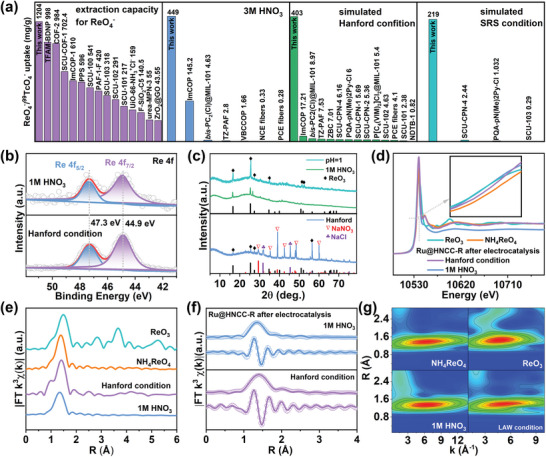
a) Comparison of the ReO_4_^−^/^99^TcO_4_^−^ extraction performance of Ru@HNCC‐R with other reported materials under various conditions. b) Re 4f XPS spectra for Ru@HNCC‐R after ReO_4_^−^ adsorption‐electrocatalysis under various conditions. c) PXRD patterns of the electrocatalytically generated products under various conditions. d) Re L_3_‐edge XANES spectra for Ru@HNCC‐R after ReO_4_^−^ adsorption‐electrocatalysis under various conditions. e,f) EXAFS curves and fitting results for Ru@HNCC‐R after ReO_4_^−^ adsorption‐electrocatalysis under various conditions. g) WT contour plots for Ru@HNCC‐R after ReO_4_^−^ adsorption‐electrocatalysis under various conditions. The reference data for ReO_3_ and NH_4_ReO_4_ in d–g were reported in our previous work.^[^ ^28^ ^]^

Next, ReO_4_^−^ extraction studies were carried out under simulated SRS and Hanford conditions. As shown in Figure 3g,h, Ru@HNCC‐R achieved ReO_4_^−^ extraction capacities of 219 and 403 mg g^−1^ in simulated SRS and Hanford solutions, respectively. In comparison, Ru@HNCC‐R as adsorbent showed much lower uptake capacity under Hanford conditions (Figure 3h). A ReO_4_^−^ extraction capacity of 203 g g^−1^ over 9 days was achieved in a large‐scale extraction in 50 L of a simulated Hanford solution, indicating Ru@HNCC‐R would be a very promising adsorbent‐electrocatalyst for use in ^99^TcO_4_^−^ capture from Hanford and SRS sites (Figure 3i). Further, the Br 3d signals disappeared after the extraction experiment in Hanford solutions, demonstrating the Br^−^ can be fully exchanged with ReO_4_^−^ (Figure S18, Supporting Information). SEM and TEM images of Ru@HNCC‐R after ReO_4_^−^ extraction (Figure S19, Supporting Information) confirmed the adsorbent‐electrocatalyst retained its initial hollow morphologies. FT‐IR spectroscopy showed that the functional groups in Ru@HNCC‐R were unchanged after electrocatalytic extraction of ReO_4_^−^ under various conditions, further confirming the good stability of Ru@HNCC‐R (Figure S20, Supporting Information). In addition, some new Re─O stretching and bending modes appeared in the spectrum at low wavenumbers after the adsorption‐electrocatalysis experiments. In comparison with other materials and technologies for ^99^TcO_4_^−^/ReO_4_^−^ extraction, the Ru@HNCC‐R adsorbent‐electrocatalyst displayed a record‐high extraction capacity and excellent selectivity under both highly acidic and basic conditions, overall properties not easily accessible using traditional physical adsorption approaches (**Figure** **4**a; and Table S5, Supporting Information).

### 2.4 Mechanism Studies

Based on the excellent ^99^TcO_4_^−^/ReO_4_^−^ extraction performance of Ru@HNCC‐R under highly acidic and basic conditions, we next applied a wide range of techniques including XPS, PXRD, XAS, and in situ Raman spectroscopy to analyse the electrocatalytic extraction products, thereby allowing a plausible mechanism for the extraction ReO_4_^−^ by adsorption‐electrocatalysis to be established. The Re 4f XPS spectrum of Ru@HNCC‐R after the ReO_4_^−^ adsorption‐electrocatalysis experiments in both 1 m HNO_3_ and Hanford solutions (Figure 4b) showed signals at 44.9 and 47.3 eV in a 4:3 area ratio, which could readily be assigned to the Re 4f_7/2_ and Re 4f_5/2_ signals, respectively, of a Re(VI) species. The PXRD pattern for the used adsorbent‐electrocatalyst showed peaks at 2θ angles of 16.57, 25.46, 27.46, 34.83, 51.07, and 52.16°, corresponding to (110), (210), (003), (310), (330), and (420) reflections, respectively, of Re(VI)O_3_ (Figure 4c). The Re L_3_‐edge X‐ray absorption near edge structure (XANES) spectrum of Ru@HNCC‐R after adsorption‐electrocatalysis was very similar to that collected for a bulk ReO_3_ standard, offering further evidence for the presence of an electrocatalytically reduced Re(VI) species (Figure 4d). The Re L_3_‐edge extended X‐ray absorption fine structure (EXAFS) spectrum and fitting results showed peaks at ≈1.4 Å, which could readily be assigned to a Re─O scattering path (similar to that expected for ReO_3_) (Figure 4e,f; and Table S6, Supporting Information). The Re L_3_‐edge wavelet transform spectra for the Re(VI) electroreduction product obtained in 1 m HNO_3_ showed only a single Re‐O scattering path centred ≈1.35 Å in R‐space and ≈6 Å in k‐space (Figure 4g), with the data being almost identical to that collected for a ReO_3_ standard. A similar result was found for the Re(VI) product obtained under the Hanford conditions (≈1.38 Å in R‐space and ≈6.2 Å in k‐space).

**Figure 5:**
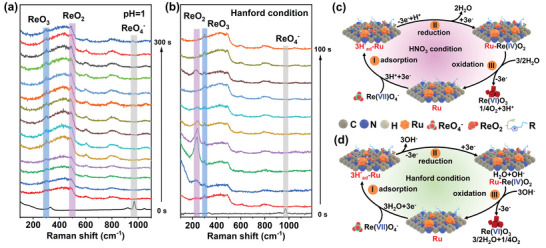
a) In situ Raman spectra collected from the Ru@HNCC‐R/carbon felt working electrode in ReO_4_^−^ solution at ≈pH 1. b) In situ Raman spectra collected from the Ru@HNCC‐R/carbon felt working electrode in ReO_4_^−^ solution under Hanford conditions. c,d) Schematic showing plausible reaction mechanisms for the Ru@HNCC‐R catalyzed extraction of ReO_4_^−^ during the adsorption‐electrocatalysis under various conditions.

We further carried out in situ Raman experiments to gain deeper understanding of the electrocatalytic processes leading to the generation of ReO_3_. In situ Raman spectra were collected from Ru@HNCC‐R during adsorption‐electrocatalytic extraction of ReO_4_^−^ form a 0.1 m HNO_3_ solution (≈pH 1) and Hanford conditions. In HNO_3_ solution, adsorbed Re(VII)O_4_^−^ was detected before electrocatalysis due to the exchange with Br^−^ in the PBA layer on Ru@HNCC‐R (**Figure** **5**a).^[^ ^26^ ^]^ During the square wave potential cycling, Raman signals at ≈500 cm^−1^ appeared that could be assigned to Re(IV)O_2_, suggesting the adsorbed Re(VII)O_4_^−^ was initially reduced to ReO_2_ (Figure S21, Supporting Information). As the reaction time increased, new Re(VI)O_3_ signals were observed at ≈300 cm^−1^. The Raman data suggests the re‐oxidation of ReO_2_ to ReO_3_, consistent with the products identified by PXRD and XAS.^[^ ^27^ ^]^ When a simulated Hanford solution was used for the tests, a ReO_2_ intermediate signals was also observed which disappeared completely at longer reaction times, consistent with the stepwise transformation of adsorbed Re(VII)O_4_^−^ to ReO_2_ and then ReO_3_ (Figure 5b).

On the basis of our findings, we propose probable mechanisms for ReO_4_^−^ extraction from acidic and alkaline solutions using the adsorption‐electrocatalysis technique (Figure 5c,d; Equations S1–S8, Supporting Information). In acidic solutions, the imidazole‐N^+^ functional sites of Ru@HNCC‐R improved surface hydrophilicity and enhanced the binding the ReO_4_^−^ (Figure 5c, step I). Simultaneously, protons adsorbed on the surface of the Ru clusters (hcp surface) forming H*_ad_‐Ru species.^[^ ^24^, ^29^ ^]^ Subsequently, the H*_ad_‐Ru reduced the adsorbed Re(VII)O_4_^−^ to a Re(IV)O_2_ intermediate under negative applied voltages (Figure 5c, step II),^[^ ^24b^ ^]^ with the desorption of water. During square wave potential cycling, Re(IV) transfers electrons to the Ru sites, with Re(VI)O_3_ being formed along with O_2_ (Figure 5c, step III). The overall equation for ReO_4_^−^ extraction under acidic conditions is thus: ReO_4_^−^ + H^+^ → ReO_3_ + 1/4O_2_ + 1/2H_2_O. A similar mechanism was developed for ReO_4_^−^ extraction under alkaline conditions. Figure 5d shows a similar electron transfer route except that H_2_O molecules adsorb at the Ru sites to form H*_ad_‐Ru and OH^−^ (step I). The electron transfer pathways through the Ru center during step II and step III under basic conditions were similar to those under acidic conditions, resulting in the overall equation: ReO_4_^−^ + 1/2H_2_O → ReO_3_ + 1/4O_2_ + OH^−^. Ru@HNCC‐R benefitted from ReO_4_^−^ specific binding sites and reversible electron transfer platform during adsorption‐electrocatalysis. Owing to the similar reduction potentials for the Re(VII)/Re(IV) and Tc(VII)/Tc(IV) redox couples, as well as the similar oxidation potentials for the Re(IV)/Re(VI) and Tc(IV)/Tc(VI) redox couples, we hypothesize that ^99^TcO_4_^−^ would be converted to ^99^TcO_2_ and then ^99^TcO_3_ via a similar reaction pathway as the electrocatalytic ReO_4_^−^ to ReO_3_ transformation process.^[^ ^30^ ^]^

Considering the above experimental evidence, the adsorption‐electrocatalysis extraction performance of Ru@HNCC‐R toward ^99^TcO_4_^−^/ReO_4_^−^ under highly acidic, strongly ionic strength, and alkaline conditions (i.e., 3 m HNO_3_, simulated Hanford and SRS conditions) is unmatched by any physiochemical adsorption technology yet developed for these two ions. Results suggest that Ru@HNCC‐R possesses many advantages for the long‐term extraction and separation of ^99^TcO_4_^−^/ReO_4_^−^ in simulated nuclear waste streams, with our adsorption‐electrocatalysis approach likely to be suitable for the selective extraction of ^99^TcO_4_^−^ (and potentially other useful metal ions) from practical nuclear waste and wastewater.

## 3 Conclusion

In summary, we have developed an adsorption‐electrocatalysis system for the efficient extraction of ^99^TcO_4_^−^/ReO_4_^−^ under extreme conditions. The adsorbent‐electrocatalyst (Ru@HNCC‐R) consists of ultrafine ruthenium clusters supported on hollow N‐doped carbon capsules whose surface was functionalized with a thin cationic polymeric network. The imidazole‐N^+^ cationic sites on the thin surface polymer layer imparted Ru@HNCC‐R with a high binding affinity toward ^99^TcO_4_^−^/ReO_4_^−^, while the Ru clusters provided a reversible electron transfer platform for the generation of ReO_3_ for easy collection. Detailed adsorption experiments revealed Ru@HNCC‐R to be an efficient adsorbent‐electrocatalyst for ^99^TcO_4_^−^ and ReO_4_^−^ ions, delivering a state‐of‐the‐art high extraction capacity, good ^99^TcO_4_^−^/ReO_4_^−^ selectivity over other common anions, and long‐term durability under highly acidic, strongly ionic strength, and alkaline conditions. The developed novel adsorption‐electrocatalysis strategy for ^99^TcO_4_^−^/ReO_4_^−^ extraction overcomes the limitations of traditional physicochemical adsorbents and other removal technologies under such extreme conditions, guiding improvements in nuclear waste management. We expect that the adsorbent‐electrocatalyst strategy reported herein will be widely adopted by other researchers for the selective removal of target anions from aqueous media.

## Conflict of Interest

The authors declare no conflict of interest.

## Supporting information

Supporting Information

## Data Availability

The data that support the findings of this study are available from the corresponding author upon reasonable request.
