# Supplementary material for: Functional Carbon Capsules Supporting Ruthenium Nanoclusters for Efficient Electrocatalytic 99TcO4 −/ReO4 − Removal from Acidic and Alkaline Nuclear Wastes
Source: Adv Sci (Weinh). 2023 Sep 10;10(30):2303536. doi: 10.1002/advs.202303536 (PMC10602505; doi:10.1002/advs.202303536)
Supplement: Supplementary file 1 — Supporting Information [file ADVS-10-2303536-s001.pdf]

ADVANCED SCIENCE Open Access

Supporting Information

for *Adv. Sci.*, DOI 10.1002/advs.202303536

Functional Carbon Capsules Supporting Ruthenium Nanoclusters for Efficient Electrocatalytic $^{99}TcO_4^-$/ReO_4^- Removal from Acidic and Alkaline Nuclear Wastes

Xiaolu Liu, Yinghui Xie, Yang Li, Mengjie Hao, Zhongshan Chen, Hui Yang, Geoffrey I. N. Waterhouse, Shengqian Ma* and Xiangke Wang**

Supporting Information

Functional Carbon Capsules Supporting Ruthenium Nanoclusters for Efficient Electrocatalytic $^{99}TcO_4^-/ReO_4^-$ Removal from Acidic and Alkaline Nuclear Wastes

Xiaolu Liu, Yinghui Xie, Yang Li, Mengjie Hao, Zhongshan Chen, Hui Yang, Geoffrey I. N. Waterhouse, Shengqian Ma,* and Xiangke Wang**

Chemicals and instrumentation

All chemicals were sourced from commercial suppliers and used without further purification. Powder X-ray diffraction (PXRD) patterns were collected on a Rigaku SmartLab SE X-ray diffractometer equipped with a Cu Kα source. BET surface areas were determined from N_2 adsorption/desorption isotherms collected at 77 K using a Micromeritics TriStar II. Scanning electron microscopy (SEM) images were recorded on Hitachi SU8010 and S4800 Scanning Electron Microscopes. Transmission electron microscopy (TEM) images, high-resolution TEM (HRTEM) images, high-angle annular dark-field scanning transmission electron microscopy (HAADF-STEM) images, energy dispersive X-ray spectroscopy (EDS) element maps, and spherical aberration corrected HAADF-STEM images were recorded on JEOL JEM-2100F or JEM-ARM200F transmission electron microscopes operating at an accelerating voltage of 200 kV. Inductively coupled plasma optical emission spectrometry (ICP-OES) analyses were performed on an Agilent 5110 spectrometer. Inductively coupled plasma mass spectrometry (ICP-MS) analyses were performed on an Agilent 7800 spectrometer system. Raman spectra were obtained from powder samples on Jobin Yvon HR-800 Raman spectrometer equipped with a Cobolt Samba single-mode 514 nm diode laser. X-ray photoelectron spectroscopy (XPS) analyses were performed using a Thermo ESCALAB 250XI spectrometer, equipped with a monochromatic Al Kα X-ray source. Fourier transform infrared spectra (FT-IR) were recorded on a SHIMADZU IRTracer-100. Water contact angle data were measured on the Germany KRUSS DSA25S instrument. Cyclic voltammograms (CV) were recorded on Metrohm Autolab and CHI 760 electrochemical workstations. Electrochemical impedance spectroscopy (EIS) tests were conducted on a CHI 760 electrochemical workstation. Electrochemical removal of ReO_4^- experiments were performed using an UNI-T UTG1005A Function/Arbitrary Waveform Generator. UV-vis single point data were recorded on a Techcomp S1020 UV-Vis spectrophotometer. $^{99}TcO_4^-$ was analyzed by a liquid scintillation counting (LSC) system (Perkin Elmer Quantulus 1220). Re L_3-edge X-ray absorption fine spectroscopy (XAFS) data were collected in transmission mode at the Shanghai Synchrotron Radiation Facility (14 W station, SSRF).

Experimental procedures

Synthesis of ZIF-8 nanocrystals

ZIF-8 nanocrystals were synthesized using a reported procedure with a slight modification.[1] In a typical synthesis, 4.1 g of 2-methylimidazole was dissolved in 60 mL of methanol (MeOH) to form a clear solution. 1.68 g of $Zn(NO_3)_2{\cdot}6H_2O$ was then added into the 2-methylimidazole solution followed by vigorous stirring for 1 h. The mixture was then incubated at room temperature without stirring. After 24 h, the product was isolated as a white powder by centrifugation and washed several times with MeOH, and finally dried overnight under vacuum.

Synthesis of ZIF-8@K-TA

In a typical synthesis, 300 mg of ZIF-8 nanocrystals were dispersed in deionized water by sonication for 10 min. Next, a tannic acid (24 mM, 10 mL) solution of ~pH 8 (adjusted by adding an aqueous 6 M KOH solution) was added to the ZIF-8 dispersion under constant stirring. After stirring for 5 min, the solid product was collected by centrifugation, washed several times with deionized water and MeOH, yielding ZIF-8@K-TA.[2]

Synthesis of ZIF-8@Ru-TA

30 mg of ruthenium chloride hydrate ($RuCl_3{\cdot}xH_2O$, >37% Ru basis) was dissolved in 150 mL of MeOH under stirring for 10 min. Subsequently, the ZIF-8@K-TA product above was added to the ruthenium chloride solution, and the resulting dispersion stirred for 3 h at room temperature. The solid was collected by centrifugation and washed several times with MeOH. Finally, the product was dried in an oven at 40 °C under vacuum to yield ZIF-8@Ru-TA.

Synthesis of Ru@HNCC

ZIF-8@Ru-TA was placed in a tube furnace and heated to 900 °C at a heating rate of 3 °C/min under an Ar atmosphere. After annealing for 3 h at 900 °C, the product was cooled to room temperature under Ar to give Ru@HNCC.

Synthesis of PBA

0.01 mol of 1-butyl-3-vinylimidazolium bromide([C_4VIm]Br), 0.04 mol of acrylonitrile (AN) and 0.02122 g of azodiisobutyronitrile (AIBN) were dissolved in 15 mL of dimethyl sulfoxide (DMSO) and the resulting solution heated to 69 °C under a N_2 atmosphere. After refluxing for 12 h, the copolymer was obtained and washed with acetone several times, then dried under vacuum at 45 °C for 12 h, yielding the final product 1-butyl-3-vinylimidazolium bromide-co-acrylonitrile copolymer (PBA) (Scheme S1).[3]

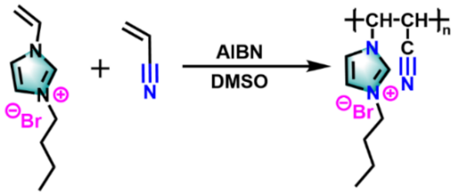

Scheme S1. Schematic diagram showing the synthesis of PBA.

Synthesis of Ru@HNCC-R

Ru@HNCC (100 mg) and PBA (100 mg) were added to 10 mL of DMSO, then the resulting dispersion heated at 40 °C under stirring until the BPA had completely dissolved. The dispersion was then dried in oven at 75 °C to obtain Ru@HNCC-R.

Materials characterization

Powder X-ray diffraction (PXRD)

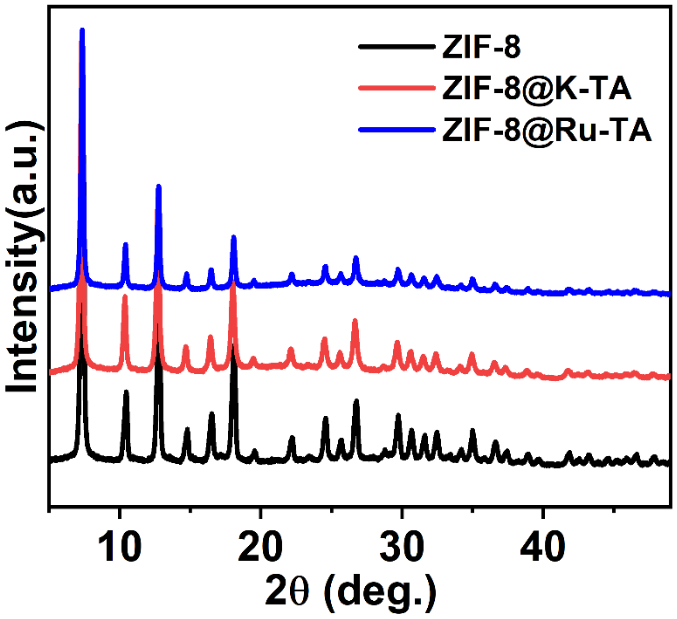

Figure S1. PXRD patterns of different materials.

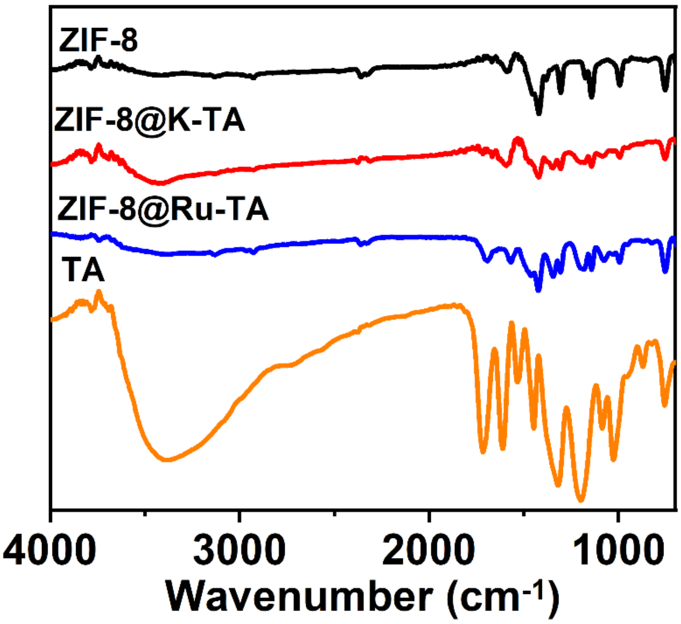

Figure S2. FT-IR spectra of different materials.

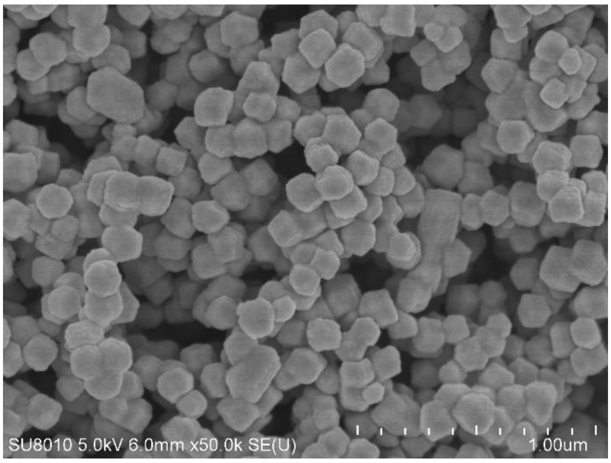

Figure S3. SEM image of ZIF-8.

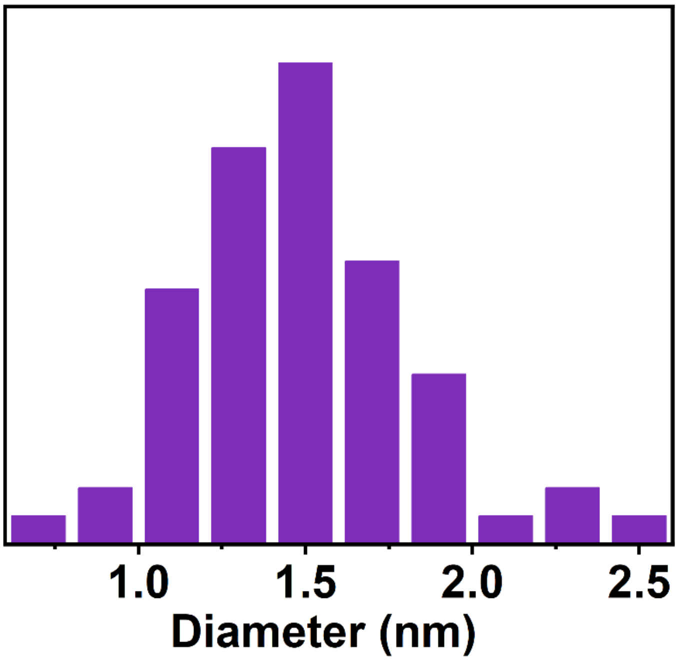

Figure S4. Particle size distribution of Ru clusters.

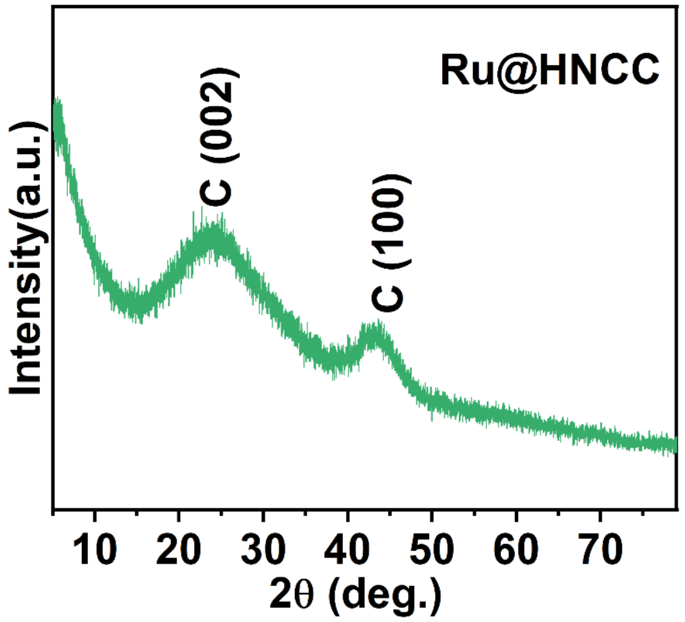

Figure S5. PXRD pattern of Ru@HNCC.

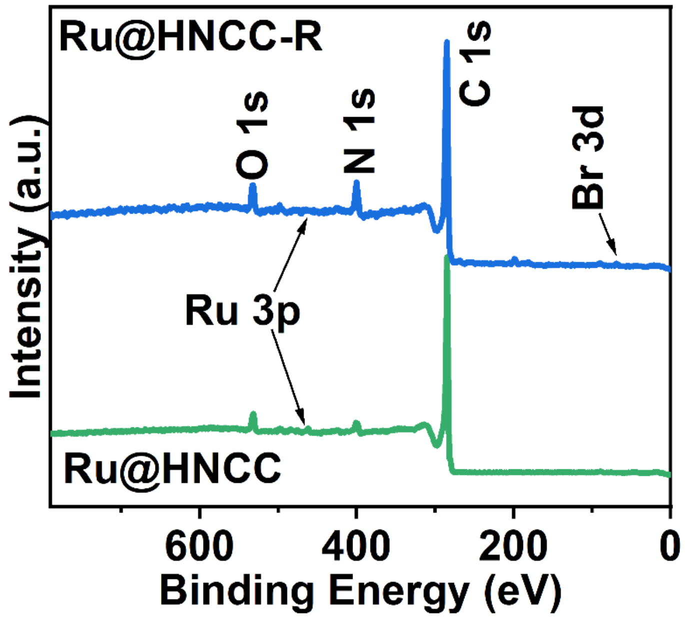

Figure S6. XPS spectra of Ru@HNCC and Ru@HNCC-R.

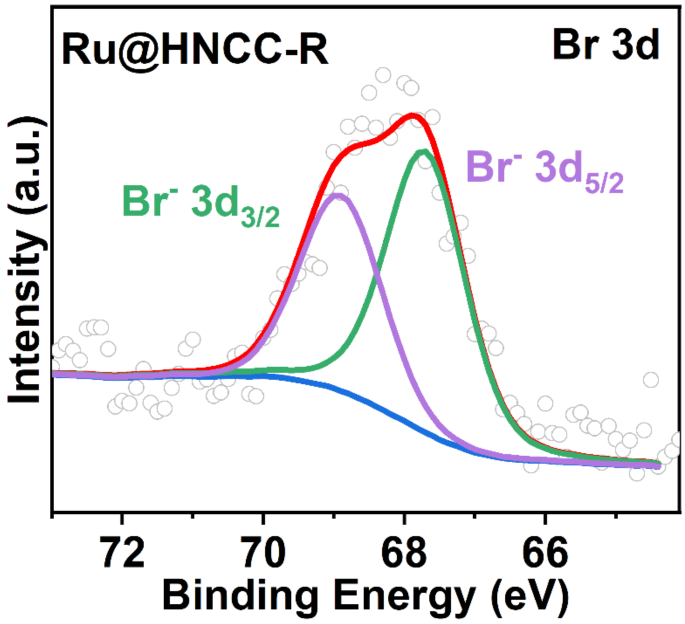

Figure S7. Br 3d XPS spectrum of Ru@HNCC-R.

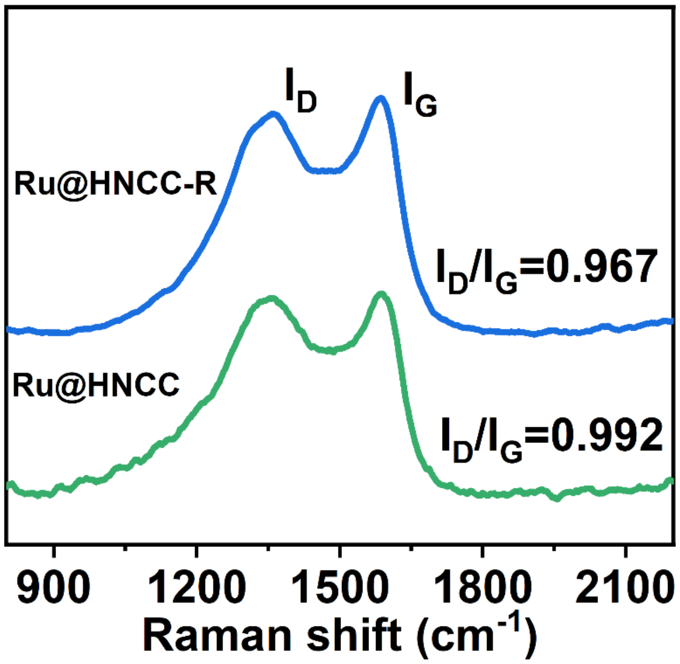

Figure S8. Raman spectra of Ru@HNCC and Ru@HNCC-R.

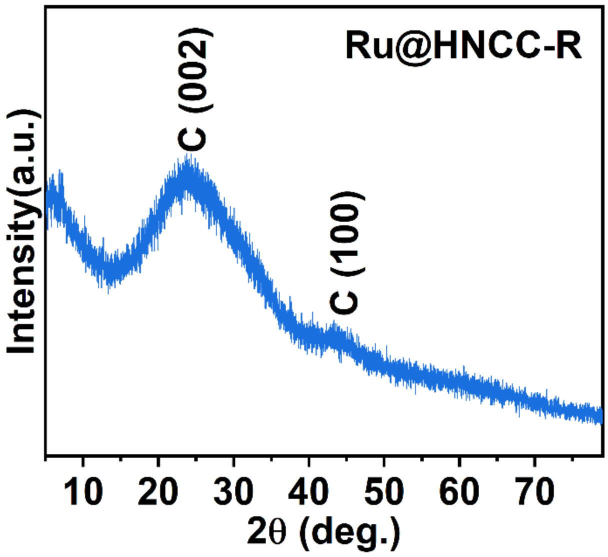

Figure S9. PXRD pattern of Ru@HNCC-R.

Table S1. Ruthenium and nitrogen contents in Ru@HNCC and Ru@HNCC-R

Material	Ru (wt.%)	N (wt.%)
Ru@HNCC	1.72	5.06
Ru@HNCC-R	0.79	7.06

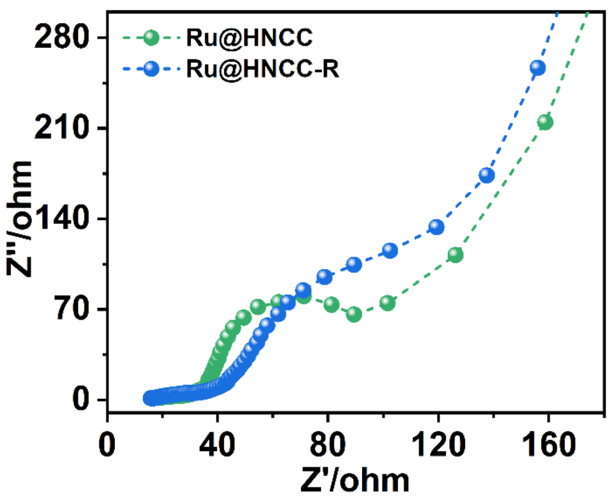

Figure S10. EIS Nyquist plots for Ru@HNCC and Ru@HNCC-R.

$^{99}TcO_4^-/ReO_4^-$ extraction experiments

$^{99}TcO_4^-$ adsorption experiments

Caution! ^{99}Tc is a β-emitter (E_{max} = 0.29 MeV). All operations relating to the handling of this substance were performed in a licensed radiochemical laboratory.

$^{99}TcO_4^-/ReO_4^-$ adsorption kinetics studies

Adsorption experiments were carried out at a fixed adsorbent/liquid ratio of 0.1 g/L at 25 °C. Ru@HNCC-R (or Ru@HNCC) was dispersed in a solution containing ~7 ppm $^{99}TcO_4^-$. Aliquots were collected at regular time intervals whilst constantly stirring the dispersion. The adsorbent was collected on a 0.22 μm membrane filter and $^{99}TcO_4^-$ in the filtrate quantified using a liquid scintillation counting (LSC) system. A solution containing ~14 ppm ReO_4^- was used to verify the $^{99}TcO_4^-$ uptake results, with the ReO_4^- concentration in the filtrate quantified spectrophotometrically at 396 nm following reaction with the chromogenic agents KSCN and $SnCl_2·2H_2O$ (chromogenic method).

ReO_4^- adsorption capacity studies

The ReO_4^- adsorption capacity experiments were carried out at 25 °C. Ru@HNCC-R (or Ru@HNCC) was added into aqueous solutions with ReO_4^- concentrations ranging from 0 to ~80 ppm at an adsorbent/liquid ratio of 0.1 g/L. The freshly prepared dispersions were sonicated and then shaken overnight. Subsequently, the dispersions were filtered on a 0.22 μm membrane filter to remove the adsorbent, with the ReO_4^- concentration in the filtrates quantified by the chromogenic method.

The ReO_4^- uptake capacity q_e (mg/g) was calculated using the following equation:

$$q_e = \frac{(C_0 - C_e) \times V}{m}$$

where C_0 and C_e are the initial concentration and equilibrium concentrations of ReO_4^- (mg/L), respectively, V is the volume of the solution (L), and m is the amount of adsorbent (g).

The isotherm data were fitted to a Langmuir model according to the following equation:

$$\frac{C_e}{q_e} = \frac{1}{K_L q_m} + \frac{C_e}{q_m}$$

where q_e is the amount of ReO_4^- adsorbed at equilibrium and C_e is the equilibrium concentration; q_m is the maximum adsorption amount; K_L is an equilibrium constant related to the binding strength.

Ion selectivity study

The effect of competing anions was studied by adding SO_4^{2-}, NO_3^-, and Cl^- with concentrations of 14 ppm, 140 ppm, or 1400 ppm into a 14 ppm ReO_4^- solution. After being stirred at a rate of 180 rpm for 24 h, the adsorbent was removed on a 0.22 μm membrane filter and ReO_4^- in the filtrate quantified by ICP-MS.

$^{99}TcO_4^-$ adsorption from simulated Hanford Low Activity Waste (LAW) Melter Recycle Stream

The simulated Hanford Low Activity Waste (LAW) Melter Recycle Stream was prepared according to a reported protocol.[4] Adsorption experiments were carried out at a fixed adsorbent/liquid ratio of 5 g/L. After stirring for 2 h, the adsorbent was collected on a 0.22 μm membrane filter and $^{99}TcO_4^-$ in the filtrate analyzed using a liquid scintillation counting (LSC) system. ReO_4^- adsorption experiments were used to verify the $^{99}TcO_4^-$ uptake results.

Electrochemical ReO_4^- extraction studies

All electrochemical ReO_4^- extraction tests were performed using a square wave conversion method employing alternating voltages between -5 and 0 V (using a frequency of 400 Hz during the tests) on a function/arbitrary waveform generator (UTG1005A). Ru@HNCC-R/carbon felt was used as the cathode and anode. The concentrations of the ReO_4^- in the electrolyte were determined by ICP-MS and a chromogenic method. After adsorption-electrocatalytic testing, the Ru@HNCC-R/carbon felt working electrode was washed with distilled water and dried under vacuum at 60 °C. Then, the electrode was subsequently returned to the electro-reactor for further adsorption-electrocatalytic tests. Eight cycles of adsorption-electrocatalysis tests were carried out on Ru@HNCC-R. The adsorption-electrocatalytic extraction of ReO_4^- from large-scale simulated Hanford solution by Ru@HNCC-R was studied under similar conditions (see below).

Large-scale extraction of ReO_4^- from simulated Hanford solution

The adsorption-electrocatalytic ReO_4^- extraction experiments were carried out in a 50 L of simulated Hanford Low Activity Waste (LAW) Melter Recycle Stream using a square wave conversion method employing alternating voltages between -5 and 0 V (using a frequency of 400 Hz during the tests) on a function/arbitrary waveform generator (UTG1005A). Ru@HNCC-R/carbon felt was used as the cathode and anode. At regular intervals, the concentration of the ReO_4^- was measured by a chromogenic method.

Table S2. Summary of the Langmuir adsorption parameters for ReO_4^- adsorption on Ru@HNCC and Ru@HNCC-R

**Materials**	**Langmuir isotherm parameters**		
	K_L	q_{ex} (mg/g)	R^2
Ru@HNCC	0.013	172.98±10.1	0.9946
Ru@HNCC-R	0.19	439.65±17.34	0.9834

q_{ex} is the maximum adsorption capacity for ReO_4^- determined in the experiment.

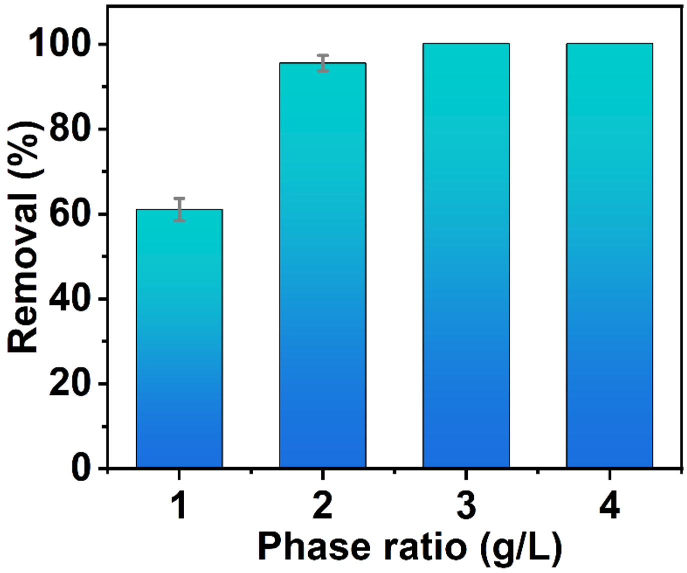

Figure S11. ReO_4^- removal by Ru@HNCC-R at various solid/liquid ratios.

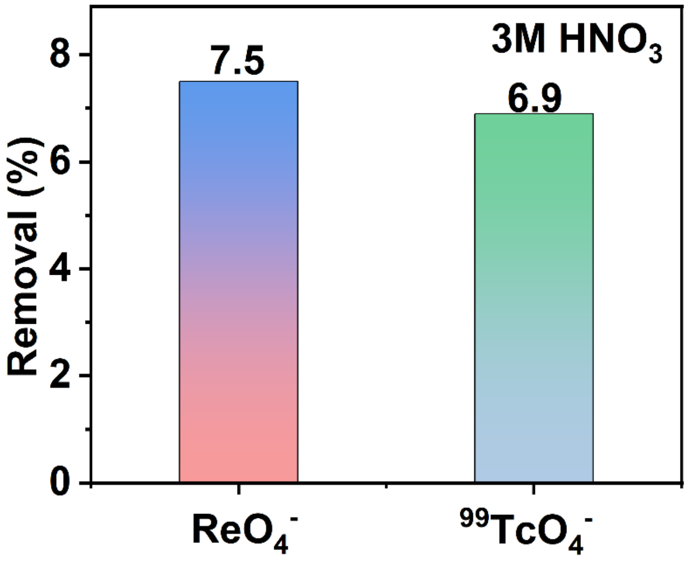

Figure S12. ReO_4^- and $^{99}TcO_4^-$ adsorption on Ru@HNCC-R in 3M HNO_3.

Table S3. Composition of Savannah River Site (SRS) High-Level Waste (HLW) Stream

Anions	Concentration (mol/L)	Molar ratio (Anion: TcO_4^-)
ReO_4^-/TcO_4^-	7.92×10^{-5}	1.0
NO_3^-	2.6	32819
OH^-	1.33	16788
NO_2^-	1.34×10^{-1}	1691
SO_2^-	6.64×10^{-6}	6576
CO_3^{2-}	4.30×10^{-5}	328

Table S4. Composition of Hanford Low Activity Waste (LAW) Melter Recycle Stream

Anions	Concentration (mol/L)	Molar ratio (Anion: TcO_4^-)
ReO_4^-/TcO_4^-	1.94×10^{-4}	1.0
NO_3^-	6.07×10^{-2}	314
Cl^-	6.39×10^{-2}	330
NO_2^-	1.69×10^{-1}	873
SO_2^-	6.64×10^{-6}	0.0343
CO_3^{2-}	4.30×10^{-5}	0.222

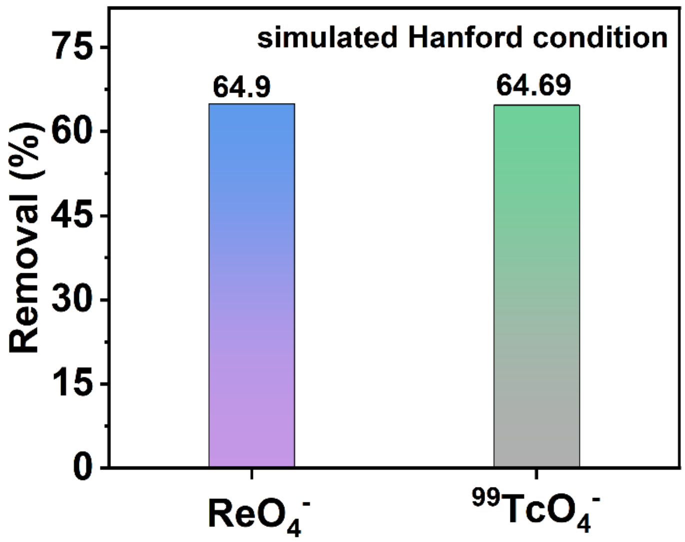

Figure S13. ReO_4^- and $^{99}TcO_4^-$ adsorption on Ru@HNCC-R under simulated Hanford conditions.

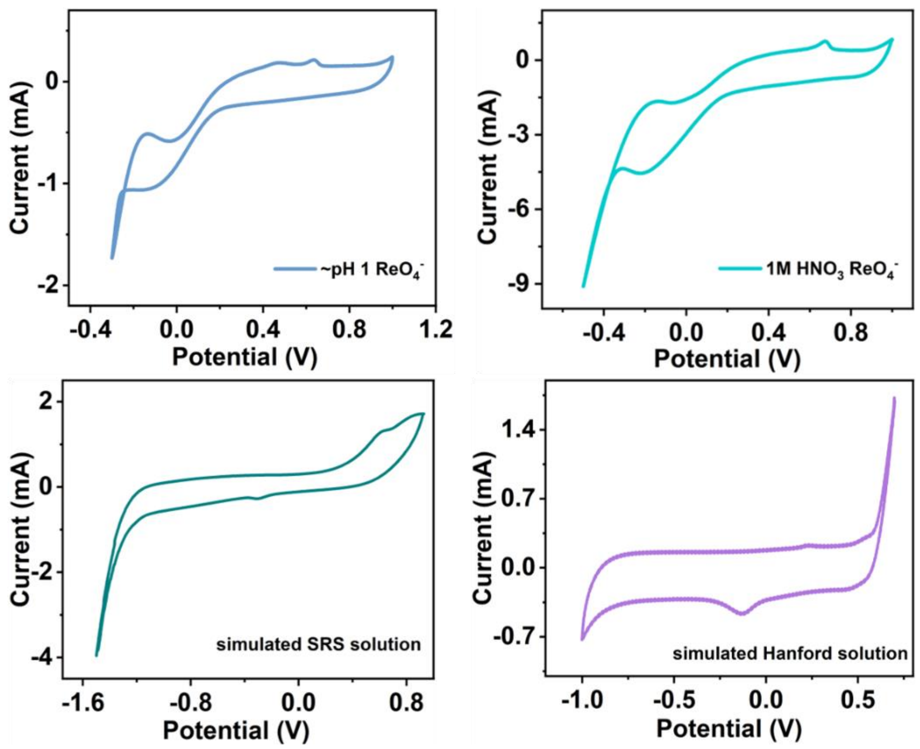

Figure S14. Cyclic voltammograms for ReO_4^- on Ru@HNCC-R under different conditions.

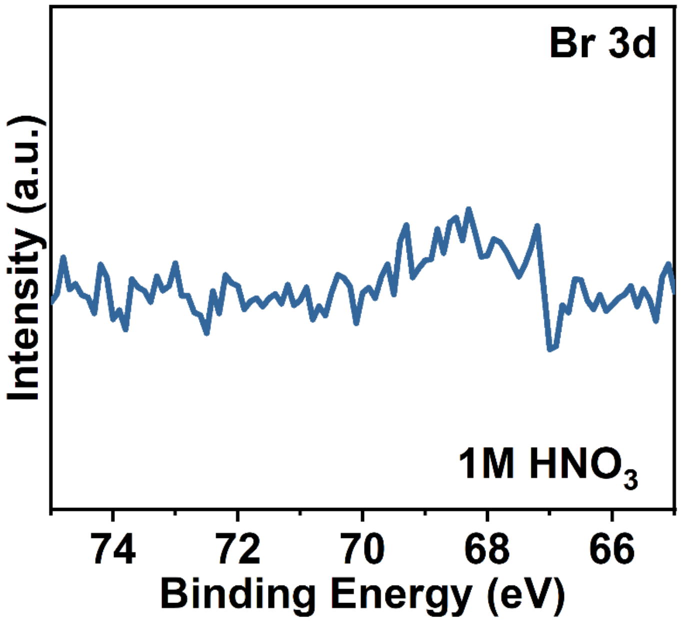

Figure S15. Br XPS spectrum for Ru@HNCC-R after adsorption-electrocatalysis in a 1M HNO_3 solution containing ~100 ppm ReO_4^-.

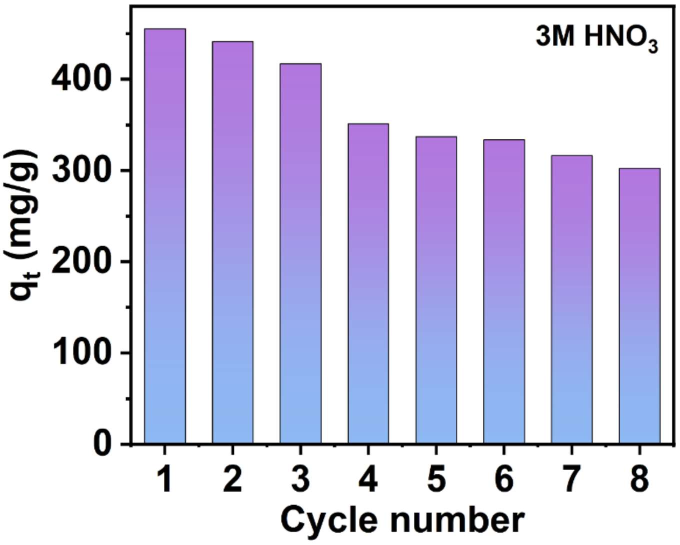

Figure S16. Recycle test data for ReO_4^- removal by Ru@HNCC-R as an adsorbent-electrocatalyst in 3M HNO_3.

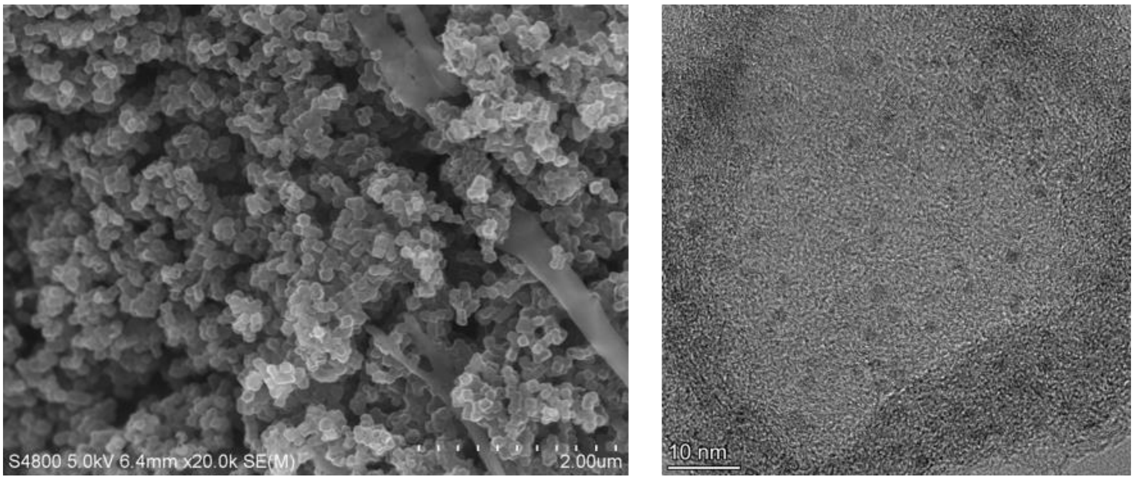

Figure S17. SEM and TEM images of Ru@HNCC-R after electrocatalytic extraction of ReO_4^- in 3M HNO_3.

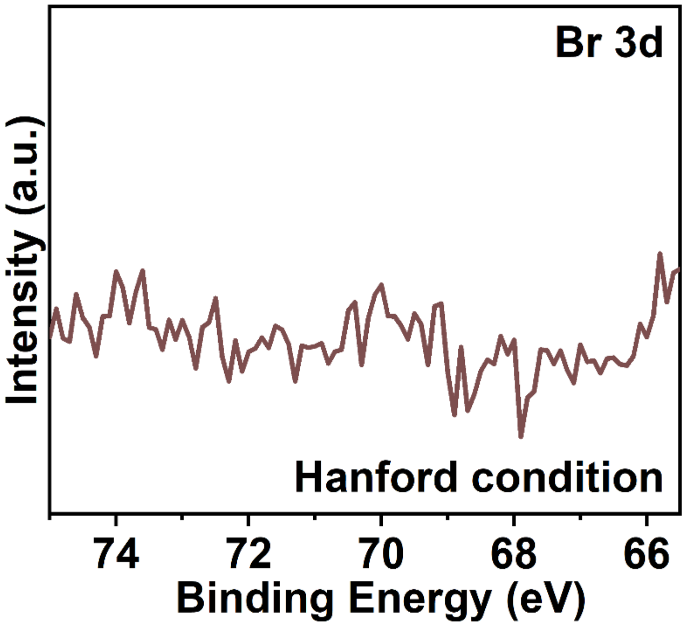

Figure S18. Br XPS spectrum for Ru@HNCC-R after adsorption-electrocatalysis under the Hanford condition.

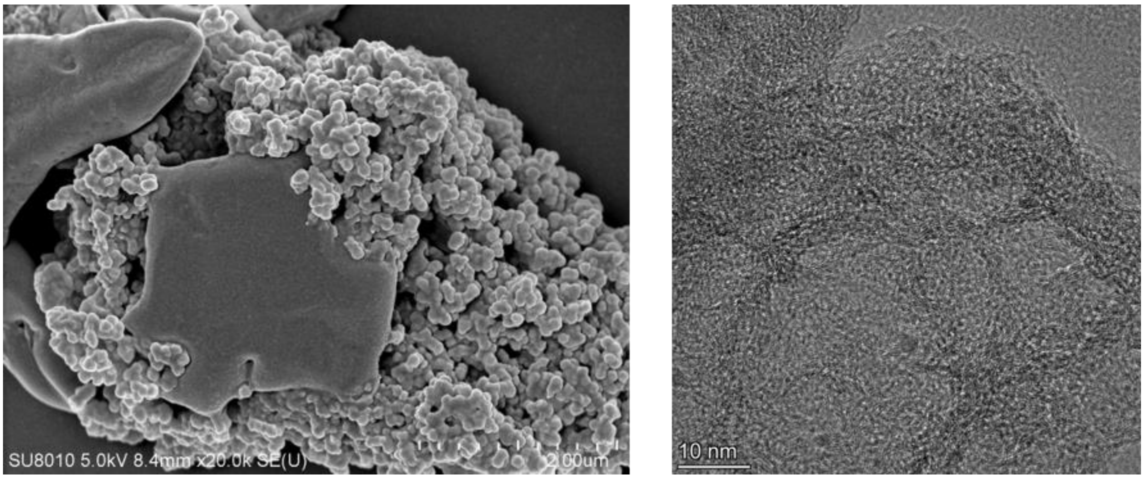

Figure S19. SEM and TEM images for Ru@HNCC-R after electrocatalytic extraction of ReO_4^- in simulated Hanford solution (50 L, 9 days).

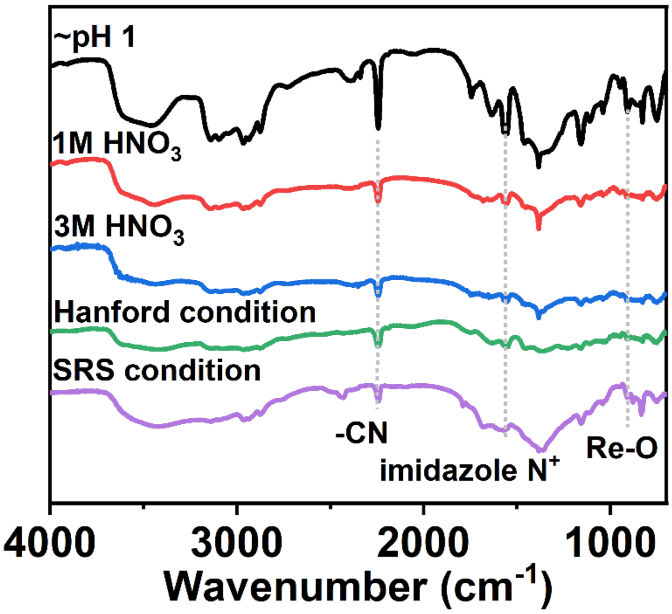

Figure S20. FT-IR spectra for Ru@HNCC-R after electrocatalytic extraction of ReO_4^- under various conditions.

Table S5. Comparison of the ReO_4^- extraction capacities of Ru@**HNCC-R and other materials**

Material	Category	Experimental conditions	Capacity (mg/g)	Ref.
**ReO_4^- solution**				
PPS	Polymer	S/L = 0.33:1, 5h	596	[5]
ImPOP-1	Polymer	S/L = 0.5:1, 24h	610	[6]
PAF-1-F	PAF	24 h	420	[7]
SCU-100	MOF	S/L=1:1, 12 h	541	[8]
SCU-101	MOF	S/L=1, 12 h	217	[9]
SCU-102	MOF	S/L=1, 12 h	291	[10]
SCU-103	MOF	m/V=1, 12 h	318	[11]
UiO-66-$NH_3^+Cl^-$	MOF	24h	159	[12]
COF-2	COF	S/L=0.4:1	984	[13]
SCU-COF-1	COF	S/L=1:1, 24 h	702.4	[14]
TFAM-BDNP	COF	S/L=0.5:1,	998	[15]
ZrO_2@rGO	Composite	S/L=0.1:1, pH= 4.0, 24 h	43.55	[16]
F-SiO_2-C5	Composite	S/L= 0.4:1, pH= 3.0, 3 h	140.5	[17]
urea-MPN-3	Polymer	S/L=1:1, pH=7	55.0	[18]
Ru@HNCC-R	Composite	S/L=0.05:1, pH=2.2	1204	This work
**3M HNO_3 solution**				
PCE fibers	PANs	S/L=50:1, C_0=25ppm 3M HNO_3	0.2825	[19]
ImCOP	Polymer	S/L=40:1, C_0=250ppm 3M HNO_3	145.2	[20]
TZ-PAF	PAFs	S/L=60:1, C_0=260ppm 3M HNO_3	2.8	[21]
NCE fibers	PANs	S/L=50:1, C_0=25ppm 3M HNO_3	0.326	[22]
bis-PC_2(Cl)@MIL-101	MOFs	S/L=100:1, C_0=463ppm 3M HNO_3	4.63	[23]
VBCOP	Polymer	S/L=60:1, C_0=181ppm 3M HNO_3	1.66	[24]
Ru@HNCC-R	Composite	S/L=0.05:1, 3M HNO_3	449	This work
**Hanford conditions**				
SCU-CPN-4	polymer	S/L=5:1 (TcO_4^-)	6.16	[25]
SCU-102	MOF	S/L=10:1	4.63	[10]
SCU-CPN-2	polymer	S/L=5:1	5.36	[26]

SCU-CPN-1	polymer	S/L=5:1 (TcO_4^-)	5.69	[27]
P[$C_4(VIM)_2$]Cl_2@MIL-101	MOF composites	S/L=5:1	5.4	[28]
bis-PC_2(Cl)@MIL-101	MOF composites	S/L=4:1	8.97	[23]
ImCOP	COP	S/L=2.5:1; S/L=5:1	17.21;9.06	[20]
TZ-PAF	PAFs	S/L=5:1	7.53	[21]
PCE fibers	PANs	S/L=10:1	4.1	[19]
NDTB-1	MOF	S/L=5:1(TcO_4^-)	0.82	[29]
SCU-101	MOF	S/L=10:1(TcO_4^-)	2.38	[9]
ZBC	Composites	S/L=5:1	7.01	[30]
PQA-pN$(Me)_2$Py-Cl	polymer	S/L=10:1 (TcO_4^-)	6	[31]
Ru@NHCC-R	Composite	S/L=0.05:1	403	This work
**SRS conditions**				
SCU-103	MOF	S/L=40:1(TcO_4^-)	0.29	[11]
SCU-CPN-4	polymer	S/L=20:1(TcO_4^-)	2.44	[25]
PQA-pN$(Me)_2$Py-Cl	polymer	S/L=10:1 (TcO_4^-)	1.032	[31]
Ru@HNCC-R	Composite	S/L=0.05:1	219	This work

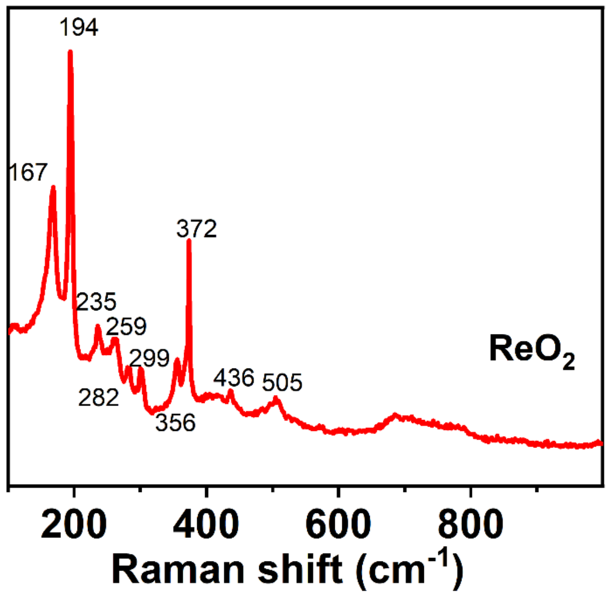

Figure S21. Raman spectrum of ReO_2.

Table S6. Summary of Re L_3-edge EXAFS curve fitting parameters after electrocatalytic reduction of ReO_4^- in 1M HNO_3 or Hanford conditions by Ru@HNCC-R.

Samples	Path	*R* (Å)	*CN*	σ^2 (Å^2)	ΔE (eV)	*R*-factor
1M HNO_3	Re-O	1.72233	3.4	0.00070	10.423	0.023
Hanford conditions	Re-O	1.73960	3.02	0.00776	9.001	0.0042

R, distance between absorber and backscattering atoms; *CN,* coordination number*;* σ^2, Debye-Waller factor to account for both thermal and structural disorders; ΔE, inner potential correction; *R-factor*, indicates the goodness of the fit.

Probable mechanisms for ReO_4^- extraction under acidic and alkaline conditions using the adsorption-electrocatalysis method

In acidic solution:

$$Ru+3H^++3e^-\rightarrow 3H^*_{ad}\text{-}Ru \quad (S1)$$

$$ReO_4^-+3H^*_{ad}\text{-}Ru+H^+\rightarrow Ru\text{-}ReO_2+2H_2O \quad (S2)$$

$$Ru\text{-}ReO_2+\frac{3}{2}H_2O\text{-}3e^-\rightarrow Ru+ReO_3+3H^++\frac{1}{4}O_2 \quad (S3)$$

$$\text{Overall reaction: } ReO_4^-+H^+\rightarrow ReO_3+\frac{1}{2}H_2O+\frac{1}{4}O_2 \quad (S4)$$

In alkaline solution:

$$Ru+3H_2O+3e^-\rightarrow 3H^*_{ad}\text{-}Ru+3OH^- \quad (S5)$$

$$ReO_4^-+3H^*_{ad}\text{-}Ru\rightarrow Ru\text{-}ReO_2+H_2O+OH^- \quad (S6)$$

$$Ru\text{-}ReO_2+3OH^-\text{-}3e^-\rightarrow Ru+ReO_3+\frac{3}{2}H_2O+\frac{1}{4}O_2 \quad (S7)$$

$$\text{Overall reaction: } ReO_4^-+\frac{1}{2}H_2O\rightarrow ReO_3+OH^-+\frac{1}{4}O_2 \quad (S8)$$

References

[1] S. R. Venna, J. B. Jasinski, M. A. Carreon, *J. Am. Chem. Soc.* **2010**, *132*, 18030-18033.

[2] a) H. Yang, S. J. Bradley, A. Chan, G. I. Waterhouse, T. Nann, P. E. Kruger, S. G. Telfer, *J. Am. Chem. Soc.* **2016**, *138*, 11872-11881; b) H. Yang, X. Wang, T. Zheng, N. C. Cuello, G. Goenaga, T. A. Zawodzinski, H. Tian, J. T. Wright, R. W. Meulenberg, X. Wang, Z. Xia, S. Ma, *CCS Chem.* **2021**, *3*, 208-218.

[3] C. Pan, L. Qiu, Y. Peng, F. Yan, *J. Mater. Chem.* **2012**, *22*, 13578-13584.

[4] J. Li, X. Dai, L. Zhu, C. Xu, D. Zhang, M. A. Silver, P. Li, L. Chen, Y. Li, D. Zuo, H. Zhang, C. Xiao, J. Chen, J. Diwu, O. K. Farha, T. E. Albrecht-Schmitt, Z. Chai, S. Wang, *Nat. Commun.* **2018**, *9*, 3007.

[5] X. Li, L. Chai, J. Ren, L. Jin, H. Wang, Y. Li, S. Ma, *Polym. Chem.* **2022**, *13*, 156-160.

[6] Z.-W. Liu, B.-H. Han, *Environ. Sci. Technol.* **2020**, *54*, 216-224.

[7] D. Banerjee, S. K. Elsaidi, B. Aguila, B. Li, D. Kim, M. J. Schweiger, A. A. Kruger, C. J. Doonan, S. Ma, P. K. Thallapally, *Chem. Eur. J.* **2016**, *22*, 17581-17584.

[8] D. Sheng, L. Zhu, C. Xu, C. Xiao, Y. Wang, Y. Wang, L. Chen, J. Diwu, J. Chen, Z. Chai, T. E. Albrecht-Schmitt, S. Wang, *Environ. Sci. Technol.* **2017**, *51*, 3471-3479.

[9] L. Zhu, D. Sheng, C. Xu, X. Dai, M. A. Silver, J. Li, P. Li, Y. Wang, Y. Wang, L. Chen, C. Xiao, J. Chen, R. Zhou, C. Zhang, O. K. Farha, Z. Chai, T. E. Albrecht-Schmitt, S. Wang, *J. Am. Chem. Soc.* **2017**, *139*, 14873-14876.

[10] D. Sheng, L. Zhu, X. Dai, C. Xu, P. Li, C. I. Pearce, C. Xiao, J. Chen, R. Zhou, T. Duan, O. K. Farha, Z. Chai, S. Wang, *Angew. Chem. Int. Ed.* **2019**, *58*, 4968-4972.

[11] N. Shen, Z. Yang, S. Liu, X. Dai, C. Xiao, K. Taylor-Pashow, D. Li, C. Yang, J. Li, Y. Zhang, M. Zhang, R. Zhou, Z. Chai, S. Wang, *Nat. Commun.* **2020**, *11*, 5571.

[12] D. Banerjee, W. Xu, Z. Nie, L. E. V. Johnson, C. Coghlan, M. L. Sushko, D. Kim, M. J. Schweiger, A. A. Kruger, C. J. Doonan, P. K. Thallapally, *Inorg. Chem.* **2016**, *55*, 8241-8243.

[13] S. Yang, J. Yin, Q. Li, C. Wang, D. Hua, N. Wu, *J. Hazard. Mater.* **2022**, *429*, 128315.

[14] L. He, S. Liu, L. Chen, X. Dai, J. Li, M. Zhang, F. Ma, C. Zhang, Z. Yang, R. Zhou, Z. Chai, S. Wang, *Chem. Sci.* **2019**, *10*, 5183-5184.

[15] X.-R. Chen, C.-R. Zhang, W. Jiang, X. Liu, Q.-X. Luo, L. Zhang, R.-P. Liang, J.-D. Qiu, *Sep. Purif. Technol.* **2023**, *312*, 123409.

[16] Y. Gao, K. Chen, X. Tan, X. Wang, A. Alsaedi, T. Hayat, C. Chen, *ACS Sustain. Chem. Eng.* **2017**, *5*, 2163-2171.

[17] H. Weng, P. Zhang, Z. Guo, G. Chen, W. Shen, J. Chen, X. Zhao, M. Lin, *ACS Appl. Mater. Interfaces* **2021**, *13*, 8249-8262.

[18] J. Shen, W. Chai, K. Wang, F. Zhang, *ACS Appl. Mater. Interfaces* **2017**, *9*, 22440-22448.

[19] R. Zhao, D. Chen, N. Gao, L. Yuan, W. Hu, F. Cui, Y. Tian, W. Shi, S. Ma, G. Zhu, *Adv. Funct. Mater.* **2022**, *32*, 2200618.

[20] Q.-H. Hu, W. Jiang, R.-P. Liang, S. Lin, J.-D. Qiu, *Chem. Eng. J.* **2021**, *419*, 129546.

[21] Y. Huang, M. Ding, J. Ding, J. Kang, Z. Yan, P. Zhao, X. Zhou, Y. Jin, S. Chen, C. Xia, *Chem. Eng. J.* **2022**, *435*, 134785.

[22] D. Chen, Z. Liu, S. Li, X. Jing, Y. Tian, W. Hu, F. Cui, R. Zhao, G. Zhu, *Chem. Eng. J.* **2023**, *452*, 139148.

[23] C.-P. Li, H.-R. Li, J.-Y. Ai, J. Chen, M. Du, *ACS Cent. Sci.* **2020**, *6*, 2354-2361.

[24] M. Ding, L. Chen, Y. Xu, B. Chen, J. Ding, R. Wu, C. Huang, Y. He, Y. Jin, C. Xia, *Chem. Eng. J.* **2020**, *380*, 122581.

[25] J. Li, B. Li, N. Shen, L. Chen, Q. Guo, L. Chen, L. He, X. Dai, Z. Chai, S. Wang, *ACS Cent. Sci.* **2021**, *7*, 1441-1450.

[26] J. Li, L. Chen, N. Shen, R. Xie, M. V. Sheridan, X. Chen, D. Sheng, D. Zhang, Z. Chai, S. Wang, *Sci. China Chem.* **2021**, *64*, 1251-1260.

[27] J. Li, X. Dai, L. Zhu, C. Xu, D. Zhang, M. A. Silver, P. Li, L. Chen, Y. Li, D. Zuo, H. Zhang, C. Xiao, J. Chen, J. Diwu, O. K. Farha, T. E. Albrecht-Schmitt, Z. Chai, S. Wang, *Nat. Commun.* **2018**, *9*, 3007.

[28] M. Huang, Z. Lou, W. Zhao, A. Lu, X. Hao, Y. Wang, X. Feng, W. Shan, Y. Xiong, *J. Hazard. Mater.* **2022**, *422*, 126871.

[29] S. Wang, P. Yu, B. A. Purse, M. J. Orta, J. Diwu, W. H. Casey, B. L. Phillips, E. V. Alekseev, W. Depmeier, D. T. Hobbs, T. E. Albrecht-Schmitt, *Adv. Funct. Mater.*, **2012**, *22*, 2241-2250.

[30] H. Hu, L. Sun, Y. Gao, T. Wang, Y. Huang, C. Lv, Y.-F. Zhang, Q. Huang, X. Chen, H. Wu, *J. Hazard. Mater.* **2020**, *387*, 121670.

[31] Q. Sun, L. Zhu, B. Aguila, P. K. Thallapally, C. Xu, J. Chen, S. Wang, D. Rogers, S. Ma, *Nat. Commun.* **2019**, *10*, 1646.
